# Phytochemical Investigation of *Chamaemelum nobile* L. and Evaluation of Acetylcholinesterase and Tyrosinase Inhibitory Activity

**DOI:** 10.3390/plants14040595

**Published:** 2025-02-15

**Authors:** Luciana Maria Polcaro, Antonietta Cerulli, Milena Masullo, Sonia Piacente

**Affiliations:** 1 Dipartimento di Farmacia, Università degli Studi di Salerno, Via Giovanni Paolo II n. 132, 84084 Fisciano, Italy; lpolcaro@unisa.it (L.M.P.); acerulli@unisa.it (A.C.); piacente@unisa.it (S.P.); 2 Ph.D. Program in Drug Discovery and Development, University of Salerno, Via Giovanni Paolo II 132, 84084 Fisciano, Italy; 3 National Biodiversity Future Center (NBFC), 90133 Palermo, Italy

**Keywords:** *C. nobile*, sesquiterpene lactones, tyrosinase, acetylcholinesterase

## Abstract

The ageing of the world population has led to an increase in the incidence of neurodegenerative diseases. In this regard, plants have become an important source of bioactive principles that are able to act on multiple targets. *Chamaemelum nobile* (L.) All. is a perennial herb of the Asteraceae family, known as Roman chamomile, less studied in the scientific literature than the more common *Matricaria chamomilla*. Flavonoids and sesquiterpene lactones represent the main secondary metabolites. Among these, nobilin and its derivatives are considered the main components. With the aim of performing a phytochemical investigation, the extract of the fresh aerial parts of *C. nobile* was firstly analysed by LC-(+)ESI/QExactive/MS/MS, which guided the isolation of 15 compounds (coumarins, glucoside derivatives, flavonoids, and germacrane-type sesquiterpene lactones) characterised by 1D and 2D NMR spectroscopy. The presence of a derivative of nobilin, never been reported before, was highlighted. Moreover, for all isolated compounds, acetylcholinesterase and tyrosinase inhibitory activity were tested by spectrophotometric assays. The results showed that the tested compounds presented interesting tyrosinase (IC_50_ values: 32.09–412.02 µM) and acetylcholinesterase inhibitory activity (IC_50_ values: 181.58–387.99 µM). In detail, apigenin 7-*O*-rutinoside (**6**) showed the highest tyrosinase and AchE inhibitory activity, with IC_50_ values of 32.09 and 181.58 µM, respectively.

## 1. Introduction

Chamomile is indeed a plant with a long history of use, particularly for its medicinal properties. The various species of chamomile, like *Matricaria chamomilla* (German chamomile) and *Chamaemelum nobile* (Roman chamomile), have been valued for their calming effects and their ability to treat a range of ailments, from digestive issues to skin irritations. The ancient Egyptians’ reverence for chamomile reflects its importance in their medicinal practices [1]. The plant association with the Sun God also underscores its symbolic importance in Egyptian culture. Chamomile popularity spread through different cultures, and its therapeutic qualities were well documented in ancient herbal texts. Today, chamomile continues to be a popular herb, often consumed as a tea, for its soothing and anti-inflammatory properties. Its gentle nature makes it a preferred remedy for stress relief, sleep problems, and minor digestive issues [2].

The two commonly used varieties with therapeutic applications, *Matricaria chamomilla* L. and *Chamaemelum nobile* L. [1], contain several classes of bioactive compounds such as triterpenoids, sesquiterpene lactones, flavonoids, coumarins, and essential oils [3]. Although *M. chamomilla* and *C. nobile* are similar in appearance, they show some differences. *M. chamomilla* L. has small white flowers and a yellow tubular petal at its centre. *C. nobile* L. contains flowers with double petals, soft stems, and has a green apple fragrance; in fact, for this reason it is also called “the apple of the ground”. In addition, it is also known as the “Physician of Plants” due to its ability to heal sick plants around it. Another difference between the two varieties is that the petals of *M. chamomilla* are turned down and have a raised conical centre, whereas the centre of *C. nobile* is flat [4]. In this work, attention was focused on *Chamaemelum nobile* L., a variety that has been studied less in the scientific literature. A phytochemical study was carried out with the aim of investigating the biological activity of *C. nobile*-specialised metabolites. The extract obtained by SLDE-Naviglio (Solid Liquid Dynamic Extraction) was analysed by high-resolution mass spectrometry (LC–ESI/HRMS) in the positive ion mode. To assign the chemical structures of the compounds detected through LC-ESI/HRMS analysis, a phytochemical investigation of the extract was conducted, leading to the isolation and structural identification of the metabolites through 1D- and 2D-NMR.

The increasing prevalence of Alzheimer’s disease (AD) and Parkinson’s disease (PD) has indeed become a major global health issue. Given the promising results from both traditional uses and scientific research, natural products continue to be an important area of focus in the development of new therapeutic agents for AD, PD, and other cognitive disorders. These compounds not only offer potential new treatments but also provide valuable leads for the design of novel drugs with fewer side effects compared to current synthetic options. Therefore, the acetylcholinesterase and tyrosinase inhibitory activity of compounds isolated from chamomile was investigated.

## 2. Results and Discussion

### 2.1. Isolation and Identification of Specialised Metabolites from C. nobile

**Figure 1:**
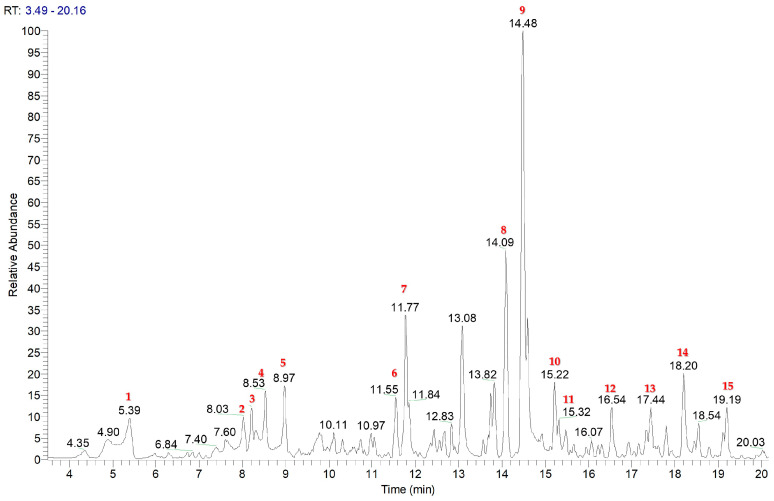
LC–(+)ESI/QExactive/MS/MS base peak profile (in positive ion mode) of *C. nobile* SLDE (50% EtOH/H_2_O) extract. The red numbers represent the compounds identified.

To obtain a chromatogram which guided the isolation of specialised metabolites, *C. nobile* SLDE-Naviglio (50% EtOH/H_2_O) extract was analysed using LC-(+)ESI/QExactive/MS/MS spectrometer operating in the positive ion mode (Figure 1). In this way, it was possible to identify 15 compounds for which an accurate analysis of the fragmentation patterns was performed and is reported in Table 1.

**Figure 2:**
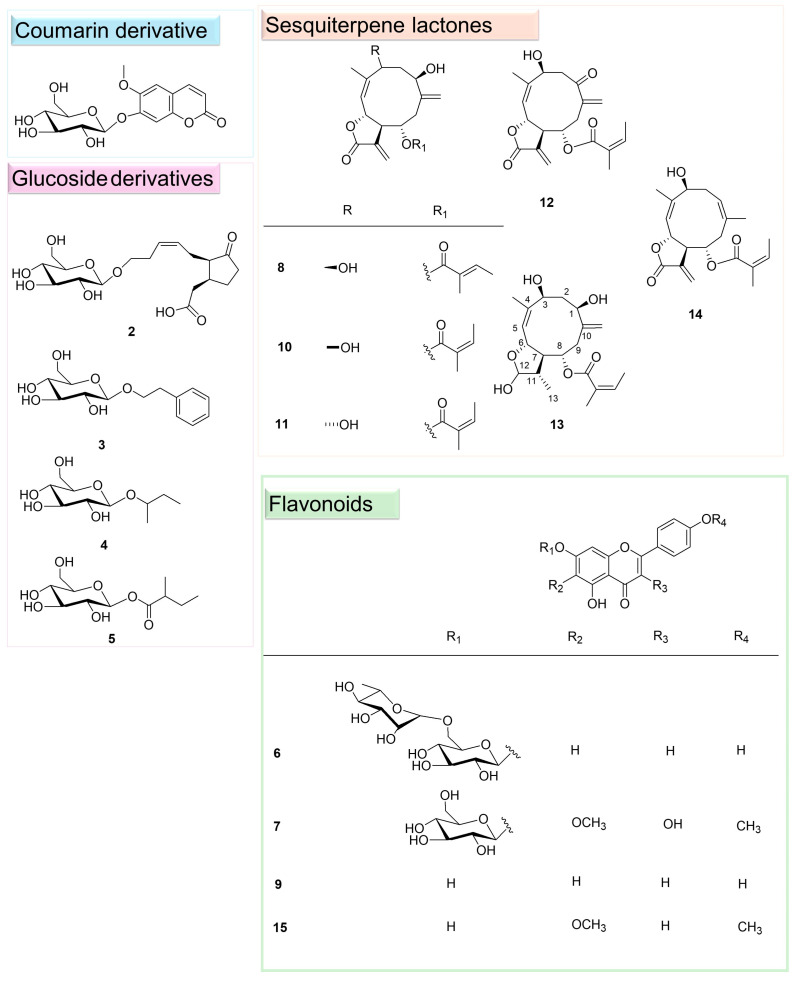
Specialised metabolites isolated from *C. nobile*.

To perform a detailed phytochemical investigation and confirm the metabolites identified by LC-ESI/HRMS analysis, the extract was fractionated on a Sephadex column, and subsequently by HPLC-UV. A total of 15 specialised metabolites (Figure 2) was isolated (Figures S1–S15). The analysis of NMR experiments (^1^H and ^13^C NMR, HSQC, HMBC, and COSY experiments) along with high-resolution MS1 and MS2 fragmentation patterns allowed us to establish their chemical structures. In detail, compound **1** belonged to coumarins; compounds **2**–**5** were characterized by a glucose moiety, and for this reason, they were grouped as glucoside derivatives. Compounds **6**, **7**, **9,** and **15** were flavonoids, while compounds **8** and **10**–**14** were elucidated as germacrane-type sesquiterpene lactones. To the best of our knowledge, compounds **3**–**5** and **7** were herein reported for the first time in *C. nobile* and the Asteraceae family. Noteworthy, compound **13** was a derivative of nobilin, never reported before.

### 2.2. Characterisation of 11,13-Dihydro-8-Tigloylhydroxyisonobilin (**13**)

The HRMS of compound **13** (*m*/*z* 387.1806 [M + Na]^+^ calculated for C_20_H_28_O_6_Na, 387.1784) and the ^13^C NMR data supported the molecular formula C_20_H_28_O_6_. The MS/MS spectrum of this compound showed a product ion at *m*/*z* 287.1254 [M + Na]^+^ (C_15_H_20_O_4_Na), corresponding to the neutral loss of 100 Da attributed to a tigloyl moiety (C_5_H_8_O_2_). The ^1^H NMR spectrum showed characteristic signals for two exomethylene protons at δ 5.55 and 5.49 (each, 1H, d, *J* = 0.5 Hz), one olefinic proton at δ 5.30 (m), four oxygen-bearing methine protons at δ 6.14 (1H, dd, *J* = 9.4, 7.8 Hz), δ 5.31 (1H, m), δ 3.93 (1H, brdd, 9.5, 5.8), δ 4.47 (1H, brt, *J* = 4.4 Hz), a secondary methyl group at δ 1.30 (3H, d, *J* = 6.8 Hz), and a tertiary methyl group at δ 1.83 (1H, s). These data, along with the analysis of 2D NMR data (HSQC, HMBC, and COSY), resembled the structure of a germacranolide skeleton similar to hydroxyisonobilin (**10**) [5], except for the replacement of an exomethylene group with a secondary methyl group (δ 1.30, Me-13). The configuration of the methyl group at C-11 was deduced to be β according to the *trans* diaxial coupling constant between H-7 and H-11 (*J* = 12.3 Hz) [6]. The NMR values and the proton coupling constant confirmed the same relative stereochemistry reported for hydroxyisonobilin (**10**) [5] (Table 2). In detail, the stereochemistry at C-l was supported by the coupling constants of H-1 (*J* = 9.5, 5.8 Hz) which indicated a β orientation for the hydroxy function [5]; the β-orientation of the C-3 hydroxy function was established on the basis of the coupling constant of H-3 (brt, *J* = 4.4 Hz) compared with that reported by De Mieri et al. [5], and the proton coupling constant of H-6 (dd, *J* = 9.4, 7.8) suggested the *trans*-fusion of the lactonic ring, as described for analogue sesquiterpene lactones [7,8].

Moreover, in the ^1^H NMR spectrum, signals at δ 6.22 (1H, brdd, *J* = 7.3, 1.3 Hz), δ 2.03 (3H, d, *J* = 7.3 Hz), and δ 1.94 (3H, brd, *J* = 1.3 Hz), corresponding to a tigloyl moiety, were evident. The HMBC correlation between H-8 (δ 5.31) with a carbon resonance at δ 167.3 revealed the linkage of the tigloyl moiety at C-8 of the germacranolide skeleton. Based on these results, compound **13** was identified as 11,13-dihydro-8-tigloylhydroxyisonobilin, which had never been reported before in the literature. ^1^H NMR, HSQC, HMBC, COSY, ^13^C NMR, and ESI-MS spectra of compound **13** have been reported in Figures S15–S20.

### 2.3. Tyrosinase Inhibition

**Figure 3:**
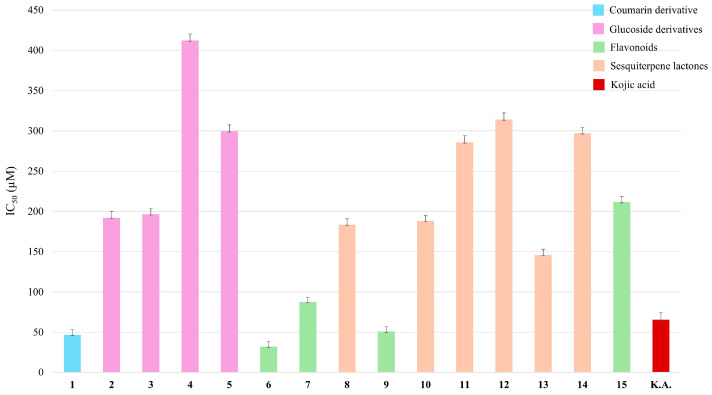
Tyrosinase inhibitory activity of specialised metabolites isolated from *C. nobile*.

The prevalence of neurocognitive disorders increases every year as the population continues to age. One of the most common neurodegenerative diseases is Parkinson’s disease (PD). Tyrosinase is one of enzyme involved in neuromelanin biosynthesis in the brain, where elevated tyrosinase activity may cause high dopamine production and, consequently, high neuromelanin production (NM) [9]. The neuromelanin accumulation disrupts neuronal proteostasis and may produce α-synuclein aggregation and PD progression. Indeed, it is well established that PD is characterised by the selective degeneration of neurons containing NM, particularly in the substantia nigra pars compacta (SNpc), leading to the hallmark motor symptoms of PD [9]. Following this evidence and considering the traditional medicinal uses of chamomile, such as inducing calmness and relaxation [3], in our ongoing investigation on tyrosinase inhibitory activity of *C. nobile* green extracts [10], for all isolated compounds, a tyrosinase inhibition assay was performed. As reported in Figure 3, flavonoids **6**, **7,** and **9** and scopolin **1** showed the highest inhibition, with IC_50_ values in a range of 32.09–50.84 μM, better than the kojic acid (65.53 μM) used as positive control. Apigenin (**9**), considered a chamomile marker [10], reported in the literature to be a natural tyrosinase inhibitor [1], showed high inhibition with IC_50_ = 50.84 µM. For the glucoside derivatives (compounds **2**–**5**), tested for the first time, the range of tyrosinase inhibition was 191.89–412.02 μM in terms of IC_50_ values. Finally, germacrane-type sesquiterpene lactones, never tested before against tyrosinase, showed IC_50_ values ranging from 145.83 to 314.10 μM, with 11,13 dihydro-8-tigloylhydroxyisonobilin (**13**) exhibiting the highest activity (IC_50_ = 145.83 µM) (Table S1).

### 2.4. Evaluation of the Inhibition Kinetic of Compound **13**

**Figure 4:**
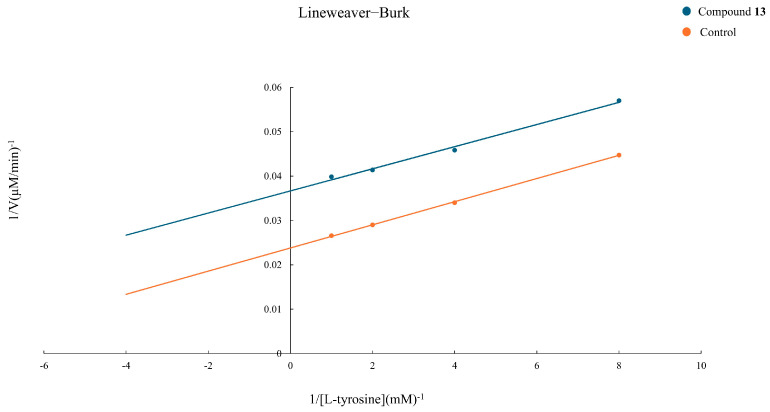
Lineweaver−Burk plots for the inhibition of compound **13** at different concentrations of L-tyrosine (1, 0.5, 0.25, and 0.125 mM) on the tyrosinase enzyme.

With the aim of understanding the type of inhibition of 11,13 dihydro-8-tigloylhydroxyisonobilin (**13**), the most active compound belonging to the class of germacrane-type sesquiterpene lactones, a kinetic study was performed. Figure 4 shows a Lineweaver–Burk double reciprocal plot for the inhibition of **13** on tyrosinase enzyme, defining uncompetitive inhibition. The maximum velocity (V_max_) value was determined as 42.02 µM/min and the Michaelis–Menten constant (K_m_) as 0.11 mM for L-tyrosine. The presence of compound **13** decreased the V_max_ value to 27.25 µM/min and the K_m_ value to 0.07 mM. These data indicated that compound **13** inhibited tyrosinase in an uncompetitive manner.

### 2.5. Acetylcholinesterase Inhibition Assay Results

Another common neurodegenerative disease is Alzheimer’s disease (AD). The main histopathological features of AD are neurofibrillary tangles (NFTs) and senile plaques, consisting of protein aggregates of the hyperphosphorylated tau protein and amyloid β (Aβ) [11]. The degeneration or atrophy of cholinergic neurons in the basal forebrain are responsible for the constant cholinergic deficit. In addition, there is a strong decrease in the neurotransmitter acetylcholine (ACh). Therefore, one therapeutic hypothesis has been to try to restore the physiological levels of ACh. Considering this, physiologically, the AChE enzyme acts by hydrolysing the neurotransmitter ACh, and the inhibition of this enzyme increases the amount of ACh present in the inter-synaptic space. Therefore, AChE inhibitors can improve cholinergic transmission by limiting the degradation of ACh. Interestingly, two of the few drugs currently licensed in Europe to alleviate cognitive symptoms in AD, galantamine and rivastigmine, are derived from natural sources. Galantamine is obtained from the bulbs of certain plants, such as *Narcissus* species (daffodils) and rivastigmine is derived from the *Alstonia* tree. Both drugs are acetylcholinesterase inhibitors, working by increasing acetylcholine levels in the brain to help alleviate the cognitive deficits seen in AD. This highlights the ongoing importance of natural products in the search for new treatments for cognitive disorders [12].

Several classes of specialised metabolites from plants have shown the ability to inhibit AChE, including coumarins, terpenes, flavonoids, glycosides, and polyphenols [13]. A large number of flavonoids (such as apigenin, biochanin, naringin, genistein, quercetin, rutin, diosmin, kaempferol-3-*O*-galactoside, luteolin-7-*O*-rutinoside, silibinin, kaempferol, quercitrin, myricetin, perlargonidin, cyanidin, epigallocatechin gallate, and chrysin) have been tested for their possible AChE inhibition, demonstrating that they possess an exceptional preclinical efficacy [14]. Also, sesquiterpene lactones have been shown to modulate cholinergic transmission by inhibiting AchE [15]. In the literature, in the last decade, multiple sesquiterpenes have been reported to possess AChE inhibitory activity [16,17,18]. As reported by Elsebay et Al., sesquiterpene lactones like amberboin and lipidiol, isolated from *Volutaria abyssinica* A. Rich (Asteraceae), inhibited AchE more than galantamine used as a reference compound [19].

**Figure 5:**
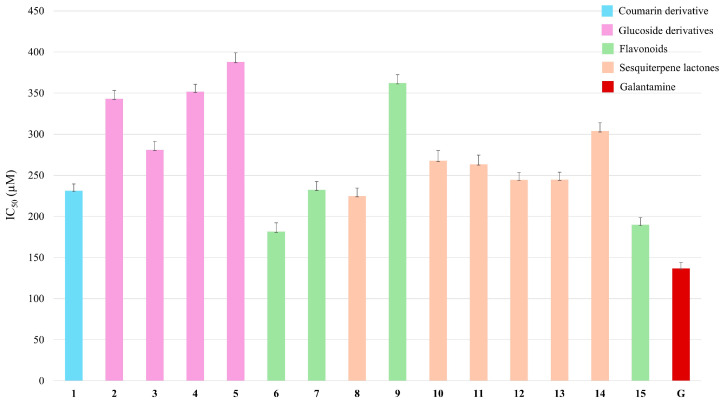
AChE inhibition assay of specialised metabolites isolated from *C. nobile*.

For these reasons, all the metabolites isolated from *C. nobile* were evaluated for their inhibition ability against the AChE enzyme. As shown in Figure 5, apigenin 7-*O*-rutinoside (**6**) showed the highest AchE inhibitory activity with an IC_50_ value of 181.58 µM, followed by compound **15** (5,7-dihydroxy-6-methoxy-2-(4-methoxyphenyl)-4H-1-benzopyran-4-one), herein tested for the first time, with an IC_50_ value of 189.82 µM. Also, scopolin (coumarin derivative, compound **1**) showed high inhibitory activity, with IC_50_ = 231.25 µM. All the germacrane-type sesquiterpene lactones, herein tested for the first time, demonstrated AChE inhibitory activity, with IC_50_ values in a range from 224.78 (**8**) to 303.71 (**14**) µM; 11,13 dihydro-8-tigloylhydroxyisonobilin (**13**) exhibited an IC_50_ value of 244.74 µM (Table S2).

## 3. Materials and Methods

### 3.1. Reagent and Solvents

MeOH and H_2_O for HPLC were purchased from VWR (Milan, Italy). CH_3_CN, HCOOH, and H_2_O for LC-MS analysis were purchased from Merck (Milan, Italy). MeOH-*d*4 (99.95%), tyrosinase enzyme from mushroom (*Agaricus bisporus*), kojic acid, L-tyrosine, acetylcholinesterase from *Electrophorus electricus* (electric eel), acetylthiocholine chloride, 5′,5′-Dithiobis-2-Nitrobenzoic Acid (DTNB), and galantamine hydrobromide were purchased from Sigma-Aldrich (Milan, Italy).

### 3.2. Sample Preparation and Extraction

Fresh plants of *Chamaemelum nobile* L. (aerial parts) were provided by the Fitomedical company (Binasco, MI, Italy), which purchased them from Azienda Agricola Bio Il Ramerino, Pitigliano (GR), a certified company in the cultivation of officinal plants [10]. The plant was cut to increase the solvent–drug contact surface, and the water content already present in the fresh plant was evaluated by calculating the dry yield. Then, it was extracted through the non-conventional extraction technique, SLDE-Naviglio, with a final mixture of 50% EtOH/H_2_O.

### 3.3. UPLC-HRMSMS Analysis

To obtain a chromatogram which guided the phytochemical study and the isolation of specialised metabolites, the extract was analysed using liquid chromatography coupled with electrospray ionisation and a high-resolution mass spectrometer (QExactive: hybrid Quadrupole-Orbitrap Mass Spectrometer, Thermo Fischer, Waltham, MA, USA), operating in the positive ion mode (for more details, see the Supporting Materials).

### 3.4. Isolation of Specialised Metabolites

*C. nobile* extract obtained via SLDE-Naviglio, using a 50% EtOH/H_2_O solution (3 g), was vacuum-dried and subjected to size exclusion chromatography on Sephadex LH-20 (25–100 μm, GE Healthcare Bio Sciences AB, Uppsala, Sweden), eluted with MeOH at a constant flow rate of 1.2 mL/min. The size of the column used was 100 × 5 cm. A total of 50 fractions were obtained and monitored by TLC (Thin Layer Chromatography). Fractions 49–50 (9.8 mg) corresponded to pure compound **9** (apigenin).

For the other fractions dissolved in MeOH (10 mg/100 μL), further purification using an RP-HPLC-UV system (Agilent Technologies 1260 Infinity, Milan, Italy) was necessary. The wavelength was set to 254 nm. The mobile phase consisted of solvent A (H_2_O + 0.1% formic acid) and solvent B (CH_3_CN + 0.1% formic acid) at a flow rate of 2 mL/min. A Sinergi 10u-Hydro RP 80A column (250 × 10.00 μm) was used. The HPLC gradient was as follows: 0–5 min from 5% to 25% B, 5–10 min from 25% to 40% B, 10–30 min from 40% to 60% B, 30–40 min from 60% to 90% B, 40–50 min from 90% to 100% B, and for 50–60 min, it was held at 100% for 5 min before returning to the starting percentage. Fraction 18 (99.5 mg) was purified to obtain compounds **4** (1.8 mg, R*_t_* = 11.2 min), **5** (1.4 mg, R*_t_* = 13.5 min), **10** (1.7 mg, R*_t_* = 36.6 min), **12** (1.0 mg, R*_t_* = 31.6 min), and the new compound **13** (1.3 mg, Rt = 37.8). Fraction 19 (108.7 mg) was chromatographed to obtain compounds **2** (0.9 mg, R*_t_* = 15.6 min), **8** (0.8 mg, R*_t_* = 35.4), **11** (0.8 mg, R*_t_* = 34.9 min), and **14** (1.1 mg, R*_t_* = 51.6). Fractions 20–21 (36.3 mg) were purified to obtain compound **3** (0.7 mg, R*_t_* = 23.2 min). Fractions 22–29 (87.4 mg) were chromatographed to obtain compound **1** (0.4 mg, R*_t_* = 15.2). Fractions 30–34 (53.7 mg) were purified to obtain compounds **6** (1.8 mg, R*_t_* = 20.1 min) and **7** (1.2 mg, R*_t_* = 24.6 min). Fractions 35–48 (67.1 mg) were purified to obtain compound **15** (0.7 mg, R*_t_* = 48.3 min).

11,13 dihydro-8-tigloylhydroxyisonobilin (**13**): white solid, [α]_D_^25^-100.00 (c 0.1, MeOH); IR (KBr) ν_max_ 3420, 1750, 1660, cm^−1^; for ^1^H NMR and ^13^C NMR data, see Table 2; HRESIMS *m*/*z* 387.1806 [M + Na]^+^ (calcd C_20_H_28_O_6_Na, 387.1784).

### 3.5. 1D and 2D NMR Analysis

For the characterisation of all isolated compounds, 1D and 2D NMR analyses were performed. For each sample, ^1^H NMR, HSQC, HMBC, and COSY spectra were acquired. For compound **13**, ^13^C NMR spectrum was carried out (for more details, see the Supporting Materials in the section General Experimental Procedures).

### 3.6. Tyrosinase Inhibition Assay

The tyrosinase inhibitory activity was evaluated using a method previously described [20,21] with slight modification (for details, see the Supporting Materials).

### 3.7. Tyrosinase Inhibition Kinetics Study

Kinetic analysis of the tyrosinase enzyme for compound **13** was carried out using the Lineweaver–Burk double reciprocal plot. The experiment was conducted at a constant enzyme concentration (100 U/mL mushroom tyrosinase) and different L-tyrosine concentrations (1, 0.5, 0.25, and 0.125 mM) in the absence (negative control) and the presence of the inhibitor (30 µL at a final concentration of 53.52 µg/mL). At 37 °C, the enzyme inhibition reaction was recorded by measuring the absorbance of the microplate reader at 495 nm for 0, 3, 6, 9, 12, and 15 min. The data obtained were plotted as 1/change in absorbance of the product (1/V) against the 1/substrate concentration (1/[S]) and V_max_ and K_m_ were calculated using the following equations: Y = 0.0026X + 0.0238 and R^2^ = 0.9996 for the control, and Y = 0.0025X + 0.0367 and R^2^ = 0.9927 for the inhibitor (compound **13**).

### 3.8. Acetylcholinesterase Inhibition Assay

The acetylcholinesterase (AchE) inhibitory activity was evaluated using a method described by Balkrishna et al. [22] with slight modification (for details, see the Supporting Materials).

## 4. Conclusions

In this work, attention was focused on *Chamaemelum nobile* L., a variety that has been little studied in the scientific literature. After a phytochemical investigation that led to the isolation of a never-reported sesquiterpene lactone, the potential CNS biological activity was evaluated for all 15 isolated compounds. Regarding the tyrosinase inhibitory activity, apigenin, apigenin 7-*O*-rutinoside, camaraside (flavonoids), and scopolin (coumarin derivative) showed the highest inhibition with IC_50_ values ranging from 32.09 to 50.84 µM. Regarding AchE inhibitory activity, the compound that showed the highest activity was 5,7-dihydroxy-6-methoxy-2-(4-methoxyphenyl)-4H-1-benzopyran-4-one, tested herein for the first time.

For a compound to be considered a potential therapeutic agent for Alzheimer’s disease (AD), one of the crucial factors is its ability to cross the blood–brain barrier (BBB) [15]. Their lipophilic nature allows sesquiterpenes to interact favourably with the lipid components of the BBB, facilitating their ability to cross it. Indeed, several studies, including in silico (computer-based) modelling, simulate how molecules interact with the BBB and cross biological membranes by passive diffusion transport [23]; in addition, within in vivo models, some of these terpenoid derivatives have demonstrated the ability to reach the CNS, highlighting their potential as therapeutic agents for AD and other neurological conditions [23].

Considering flavonoids, within the subclass of flavones (of which apigenin is a part), the flavone aglycons have been shown to have the ability to pass across the BBB [23,24]. Therefore, the specialised metabolites isolated from *C. nobile* may be interesting candidates for further studies on their potential activities on the central nervous system.

## Figures and Tables

**Table 1:** Metabolites identified in *C. nobile* SLDE (50% EtOH/H_2_O) extract by LC-(+)ESI/QExactive/MS/MS analysis.

	*R_t_*	[M + H]^+^	[M + Na]^+^	Mol Formula	Δppm	MS/MS	Name
**1**	6.97		377.0841	C_16_H_18_O_9_	−0.78	359.0734 (C_16_H_16_O_8_Na), 215.0527 (C_7_H_12_O_6_Na)	Scopolin
**2**	7.66		411.1619	C_18_H_28_O_9_	−1.64	249.1098 (C_12_H_18_O_4_Na), 203.0526 (C_6_H_12_O_6_Na)	Tuberonic acid glucoside
**3**	8.43		307.1150	C_14_H_20_O_6_	−0.93	232.0700 (C_11_H_13_O_4_Na)	Phenylethyl ꞵ-D-glucopyranoside
**4**	8.56	195.0875		C_7_H_14_O_6_	2.38	nf	1-Methylpropyl ꞵ-D-glucopyranoside
**5**	8.97		299.1122	C_12_H_20_O_7_	−2.76	nf	ꞵ-D-Glucopyranose, 1-[(2 Z)-2 methyl-2-butenoate]
**6**	10.33		601.1525	C_27_H_30_O_14_	−0.53	331.0998 (C_12_H_20_O_9_Na), 167.0704 (C_9_H_11_O_3_)	Apigenin 7-*O*-rutinoside
**7**	11.56	493.1334		C_23_H_24_O_12_	−1.37	331.080 (C_17_H_15_O_7_), 316.0577 (C_16_H_12_O_7_)	Camaraside
**8**	14.20		385.1616	C_20_H_26_O_6_	−0.59	303.1209 (C_15_H_20_O_5_Na), 285.1098 (C_15_H_18_O_4_Na)	8-Tigloylhydroxyisonobilin
**9**	14.52	271.0595		C_15_H_10_O_5_	−2.51	119.0492 (C_8_H_7_O)	Apigenin
**10**	16.64		385.1616	C_20_H_26_O_6_	−0.72	303.1209 (C_15_H_20_O_5_Na), 285.1098 (C_15_H_18_O_4_Na)	Hydroxyisonobilin
**11**	16.66		385.1619	C_20_H_26_O_6_	−0.83	303.1209 (C_15_H_20_O_5_Na), 285.1098 (C_15_H_18_O_4_Na)	3-Epi-hydroxyisonobilin
**12**	17.49		383.1459	C_20_H_24_O_6_	−0.44	283.0945 (C_15_H_16_O_4_Na), 239.1050 (C_14_H_16_O_2_Na)	Nobilinon A
**13**	17.85		387.1806	C_20_H_28_O_6_	−1.07	287.1254 (C_15_H_20_O_4_Na)	11,13-Dihydro-8-tigloylhydroxyisonobilin
**14**	17.90		369.1671	C_20_H_26_O_5_	0.84	269.1140 (C_15_H_18_O_3_Na), 251.1039 (C_15_H_16_O_2_Na)	Nobilin
**15**	19.19	315.0862		C_17_H_14_O_6_	−1.49	300.0627 (C_16_H_12_O_6_)	5,7-Dihydroxy-6-methoxy-2-(4-methoxyphenyl)-4H-1-benzopyran-4-one

**Table 2:** ^1^H and ^13^C NMR (600 and 150 MHz) of compound **13** in CD_3_OD (δ in ppm).

**13**		
	**δ_H_ (*J* in Hz)**	**δc**
1	3.93 (brdd, 9.5, 5.8)	73.5 CH
2	2.28 (m), 2.25 (m)	38.0 CH_2_
3	4.47 (brt, 4.4)	73.0 CH
4	-	144.0 C
5	5.30 (m)	125.9 CH
6	6.14 (dd, 9.4, 7.8)	76.5 CH
7	2.32 (m)	55.1 CH
8	5.31 (m)	77.3 CH
9	2.94 (dd, 14.8, 2.3) 2.35 (m)	39.7 CH_2_
10	-	145.5 C
11	2.60 (dq, 12.3, 7.2)	40.5 CH
12	-	180.0 C
13	1.30 (d, 6.8)	16.7 CH_3_
14	5.55 (d, 0.5) 5.49 (d, 0.5)	117.8 CH_2_
15	1.83 (s)	23.5 CH_3_
16	-	167.3 C
17	-	128.0 C
18	6.22 (brdd, 7.3, 1.3)	139.1 CH
19	1.94 (brd, 1.3)	20.3 CH_3_
20	2.03 (d, 7.3)	15.7 CH_3_

## Data Availability

The data presented in this study are available in the main article and in the Supplementary Materials.

## Supplementary Materials

The following supporting information can be downloaded at https://www.mdpi.com/article/10.3390/plants14040595/s1, Figures S1–S14: ^1^H NMR spectra (600 MHz, CD_3_OD) of compound **1**–**12**, **14**, and **15;** Figures S15–S19: ^1^H NMR, HSQC, HMBC, COSY, and 13C NMR spectra (600 MHz, CD_3_OD) of compound **13**; Figure S20: ESI-MS of compound **13**, Table S1: Tyrosinase inhibition activity of specialised metabolites isolated from *C. nobile*, Table S2: AchE inhibition activity of specialised metabolites isolated from *C. nobile*.
