# Supplementary material for: Phytochemical Investigation of Chamaemelum nobile L. and Evaluation of Acetylcholinesterase and Tyrosinase Inhibitory Activity
Source: Plants (Basel). 2025 Feb 15;14(4):595. doi: 10.3390/plants14040595 (PMC11858884; doi:10.3390/plants14040595)
Supplement: Supplementary file 1 [file plants-14-00595-s001.zip › plants-3462529-supplementary.pdf]

## Supporting Materials

# Phytochemical investigation of *Chamaemelum nobile* L. and evaluation of acetylcholinesterase and tyrosinase inhibitory activity

Luciana Maria Polcaro[1], Antonietta Cerulli [1], Milena Masullo[1,*], and Sonia Piacente[1,3]

[1] Dipartimento di Farmacia, Universita` degli Studi di Salerno, Via Giovanni Paolo II, 84084, Salerno, Italy
[2] PhD Program in Drug Discovery and Development, Università degli Studi di Salerno, via Giovanni Paolo II n. 132, I-84084 Fisciano, SA, Italy.
[3] National Biodiversity Future Center (NBFC), Palermo, Italy
* Correspondence: mmasullo@unisa.it; Tel.:+39 089969763; Fax:+39 089969602

**List of Supporting Materials figures and tables**

**Figure S1.** $^1$H NMR spectrum (600 MHz, $CD_3OD$) of compound **1**

**Figure S2.** $^1$H NMR spectrum (600 MHz, $CD_3OD$) of compound **2**

**Figure S3.** $^1$H NMR spectrum (600 MHz, $CD_3OD$) of compound **3**

**Figure S4.** $^1$H NMR spectrum (600 MHz, $CD_3OD$) of compound **4**

**Figure S5.** $^1$H NMR spectrum (600 MHz, $CD_3OD$) of compound **5**

**Figure S6.** $^1$H NMR spectrum (600 MHz, $CD_3OD$) of compound **6**

**Figure S7.** $^1$H NMR spectrum (600 MHz, $CD_3OD$) of compound **7**

**Figure S8.** $^1$H NMR spectrum (600 MHz, $CD_3OD$) of compound **8**

**Figure S9.** $^1$H NMR spectrum (600 MHz, $CD_3OD$) of compound **9**

**Figure S10.** $^1$H NMR spectrum (600 MHz, $CD_3OD$) of compound **10**

**Figure S11.** $^1$H NMR spectrum (600 MHz, $CD_3OD$) of compound **11**

**Figure S12.** $^1$H NMR spectrum (600 MHz, $CD_3OD$) of compound **12**

**Figure S13.** $^1$H NMR spectrum (600 MHz, $CD_3OD$) of compound **14**

**Figure S14.** $^{1}$H NMR spectrum (600 MHz, $CD_3OD$) of compound **15**

**Figure S15.** $^{1}$H NMR spectrum (600 MHz, $CD_3OD$) of compound **13**

**Figure S16.** HSQC spectrum ($CD_3OD$) of compound **13**

**Figure S17.** HMBC spectrum ($CD_3OD$) of compound **13**

**Figure S18.** COSY spectrum ($CD_3OD$) of compound **13**

**Figure S19.** $^{13}$C NMR spectrum (150 MHz, $CD_3OD$) of compound **13**

**Figure S20.** ESI-MS of compound **13**

**Table S1.** Tyrosinase inhibition activity of specialized metabolites isolated from *C. nobile*

**Table S2.** AchE inhibition activity of specialized metabolites isolated from *C. nobile*

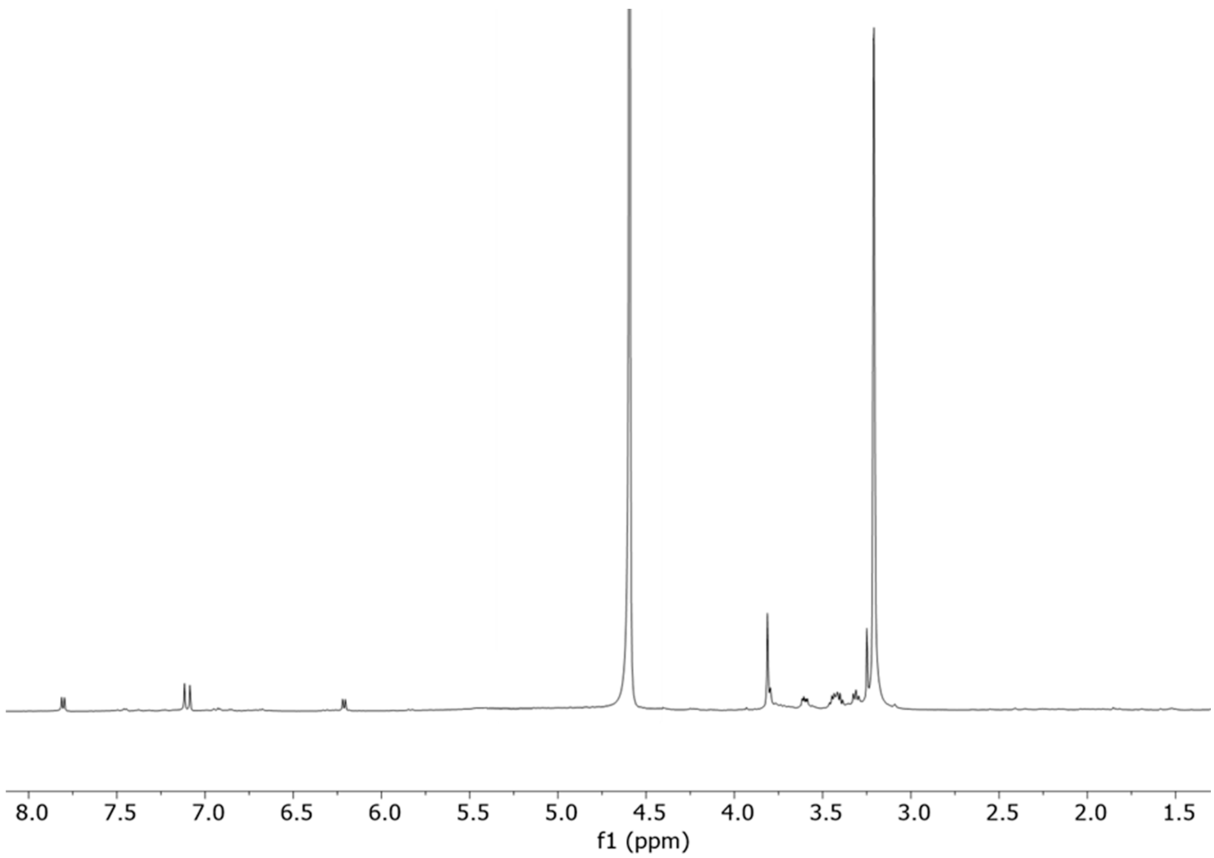


**Figure S1.** $^{1}H$ NMR spectrum (600 MHz, $CD_3OD$) of compound **1**

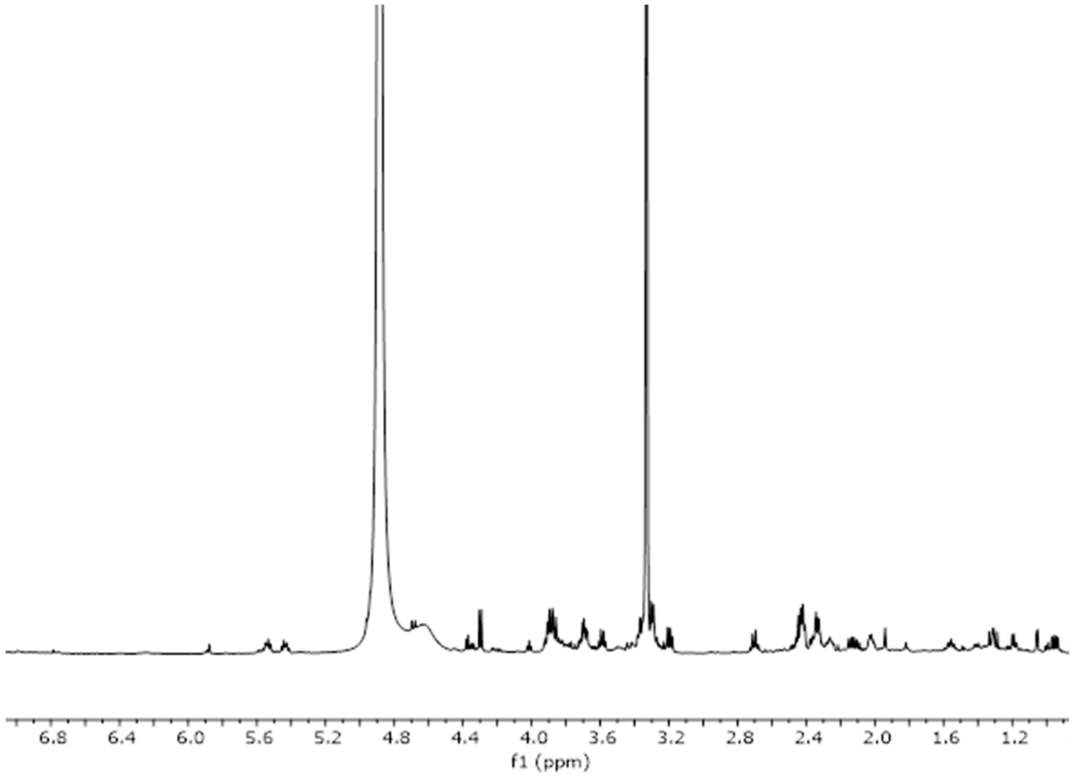


**Figure S2.** $^{1}H$ NMR spectrum (600 MHz, $CD_3OD$) of compound **2**

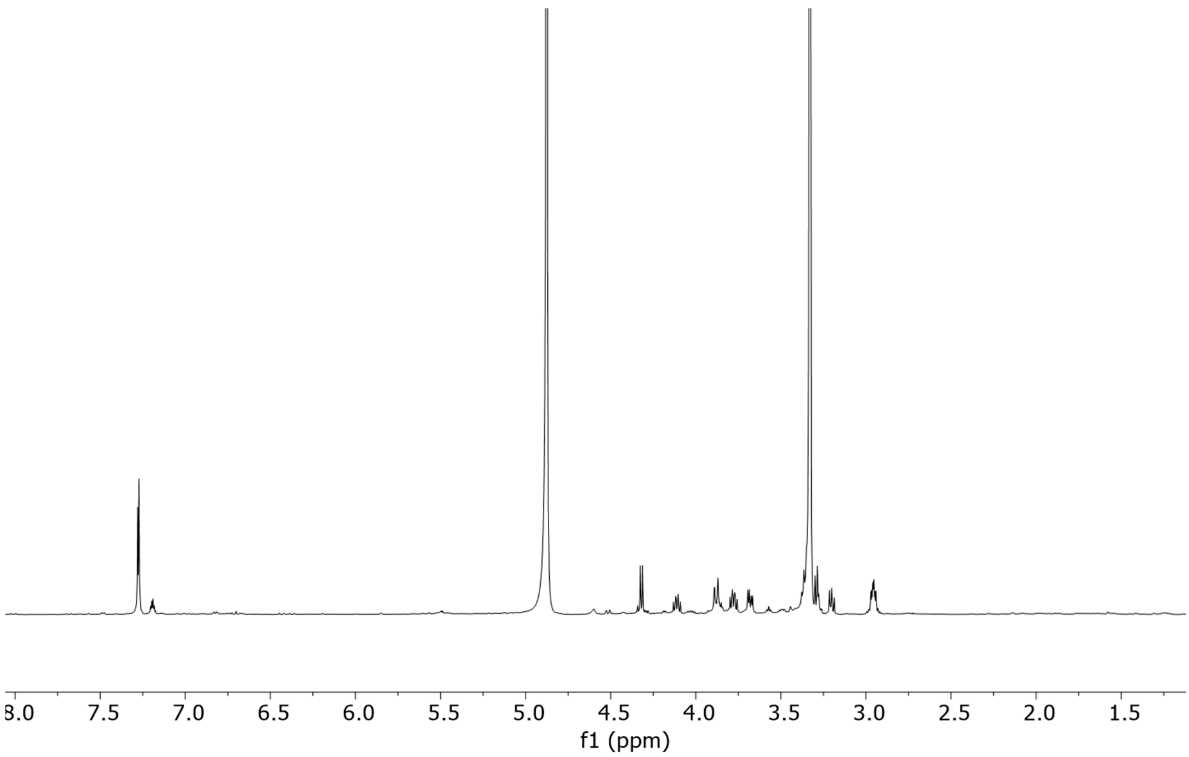


**Figure S3.** $^{1}H$ NMR spectrum (600 MHz, $CD_3OD$) of compound **3**

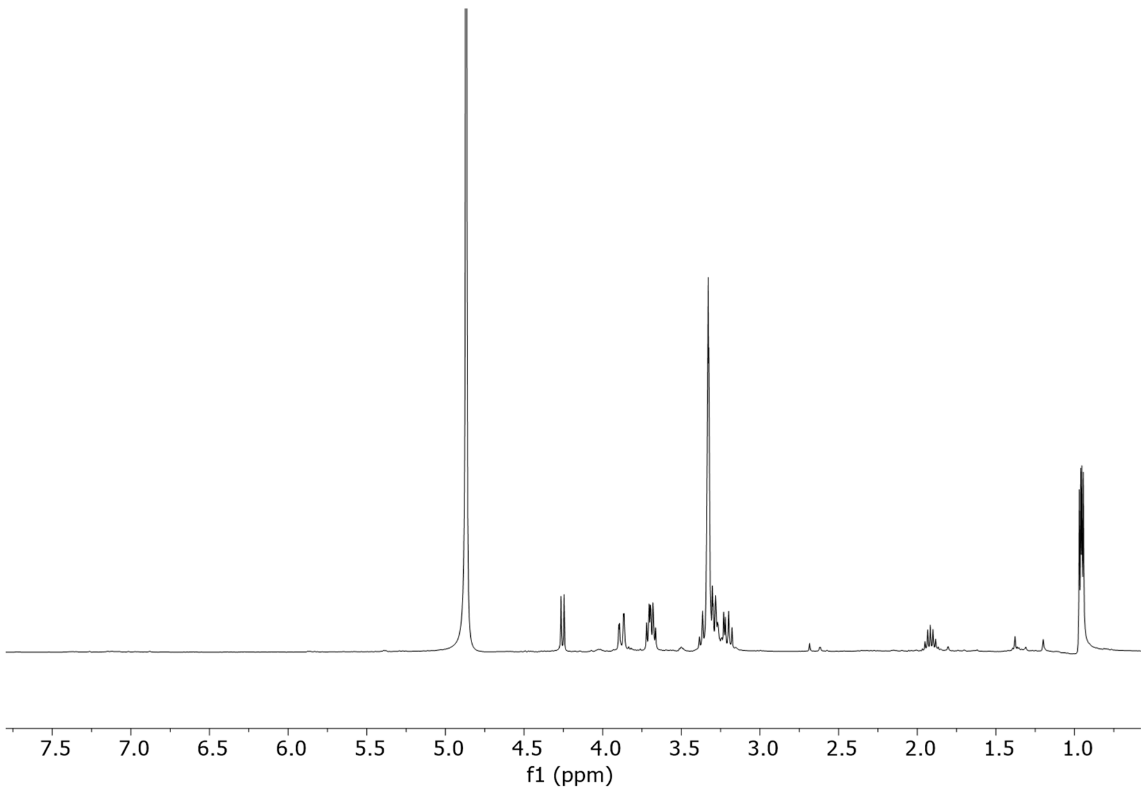


**Figure S4.** $^{1}H$ NMR spectrum (600 MHz, $CD_3OD$) of compound **4**

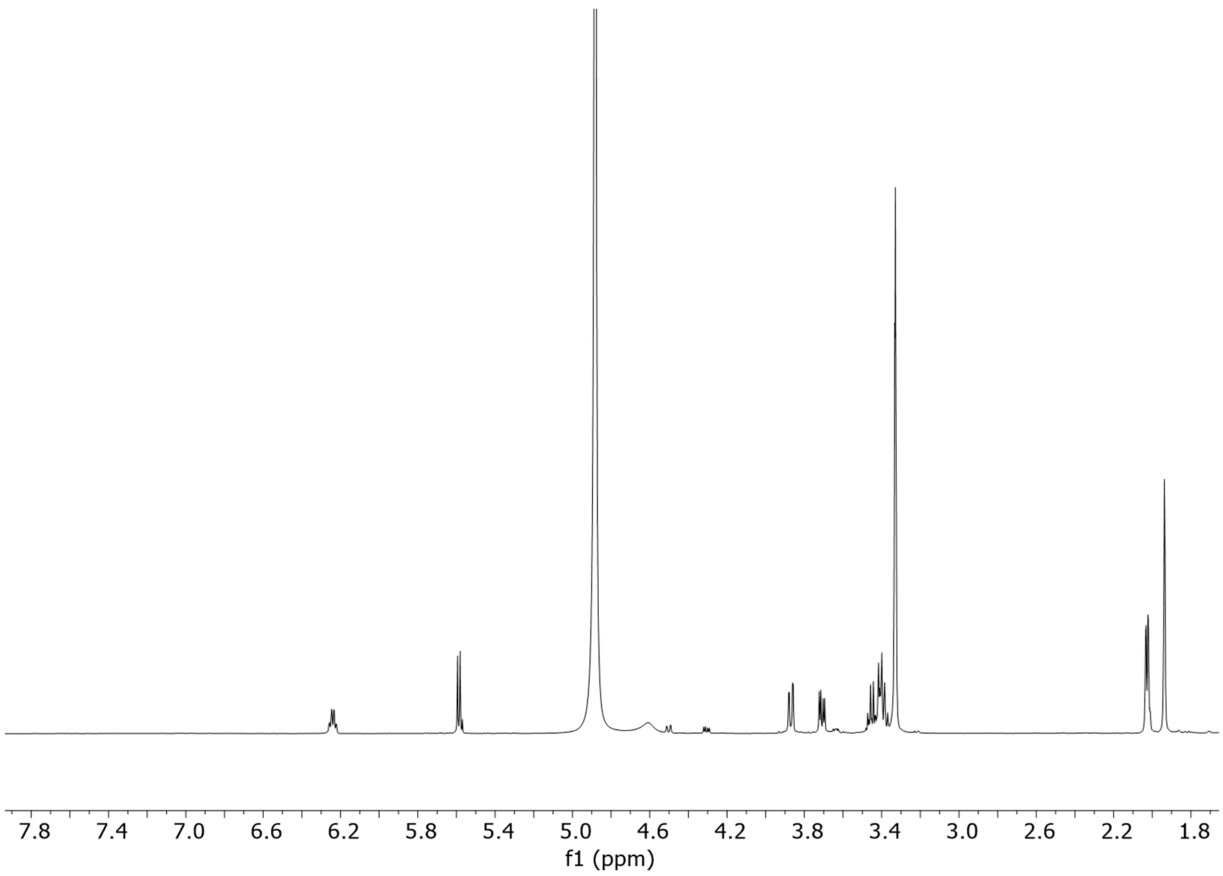


**Figure S5.** $^{1}$H NMR spectrum (600 MHz, $CD_3OD$) of compound **5**

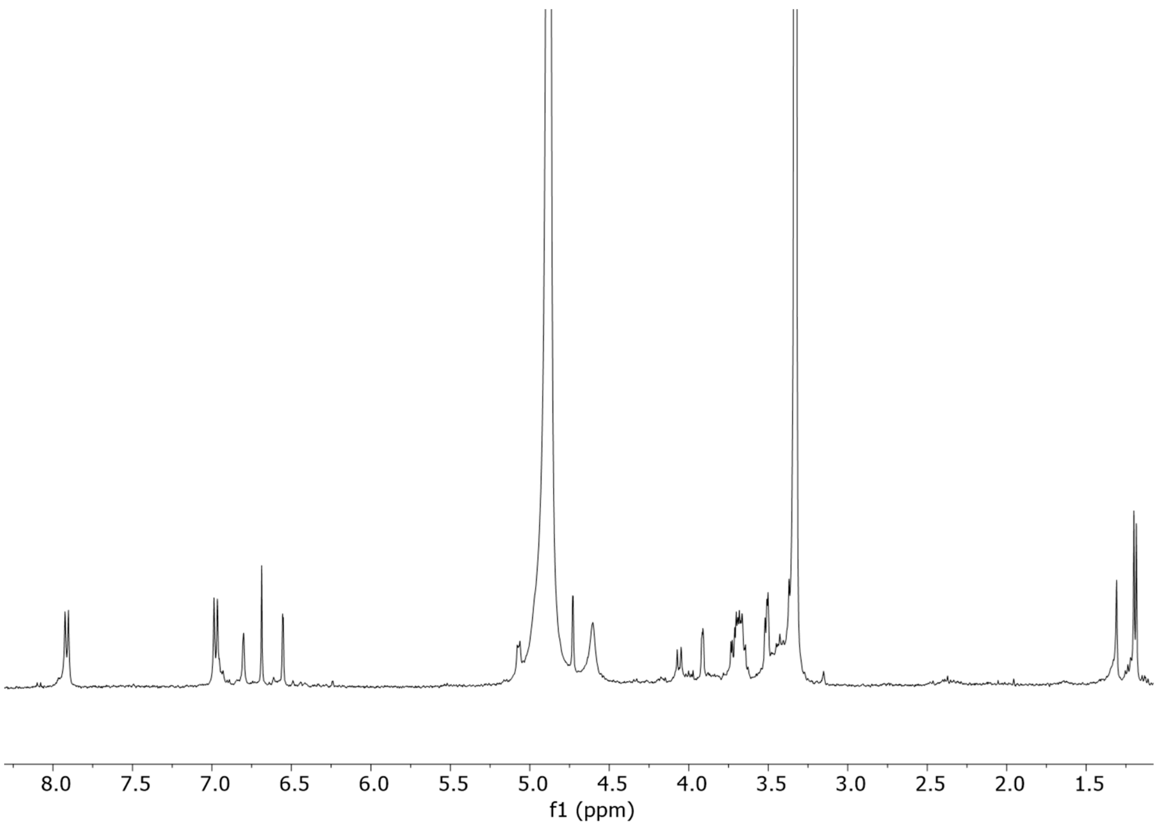


**Figure S6.** $^{1}$H NMR spectrum (600 MHz, $CD_3OD$) of compound **6**

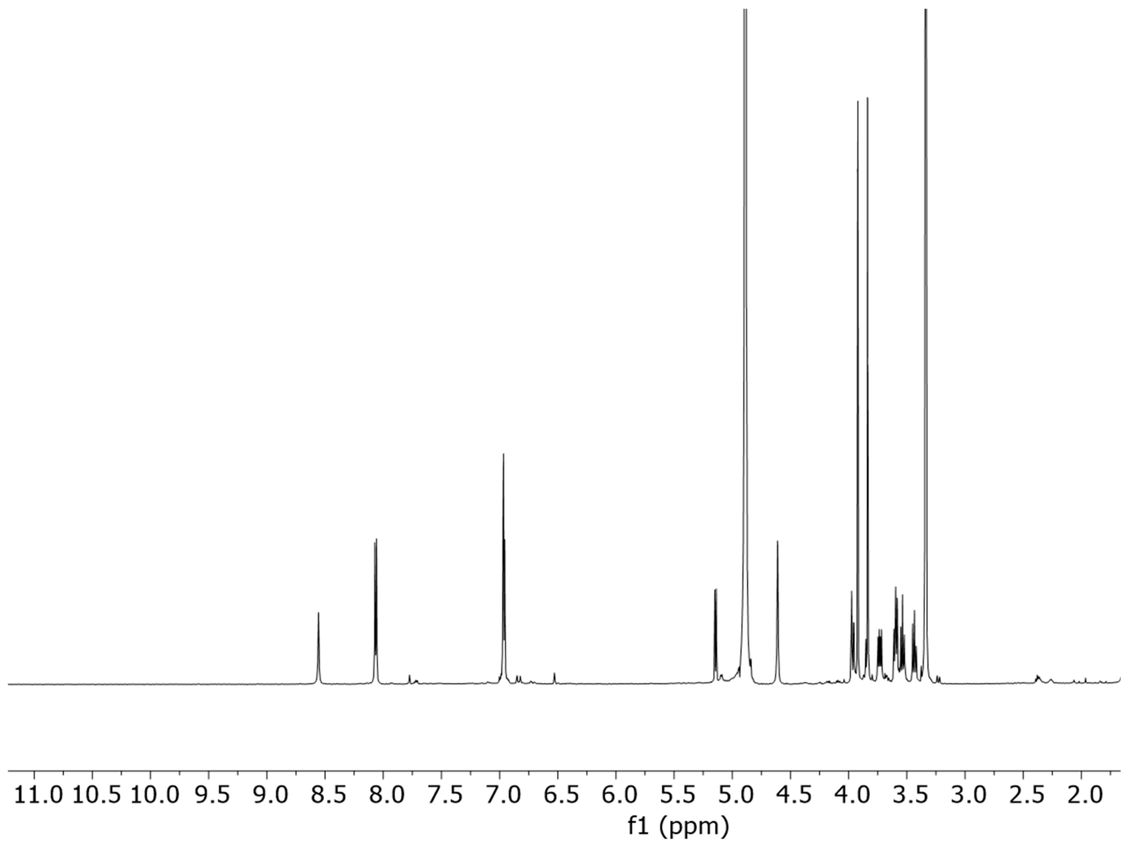


**Figure S7.** $^1$H NMR spectrum (600 MHz, $CD_3OD$) of compound **7**

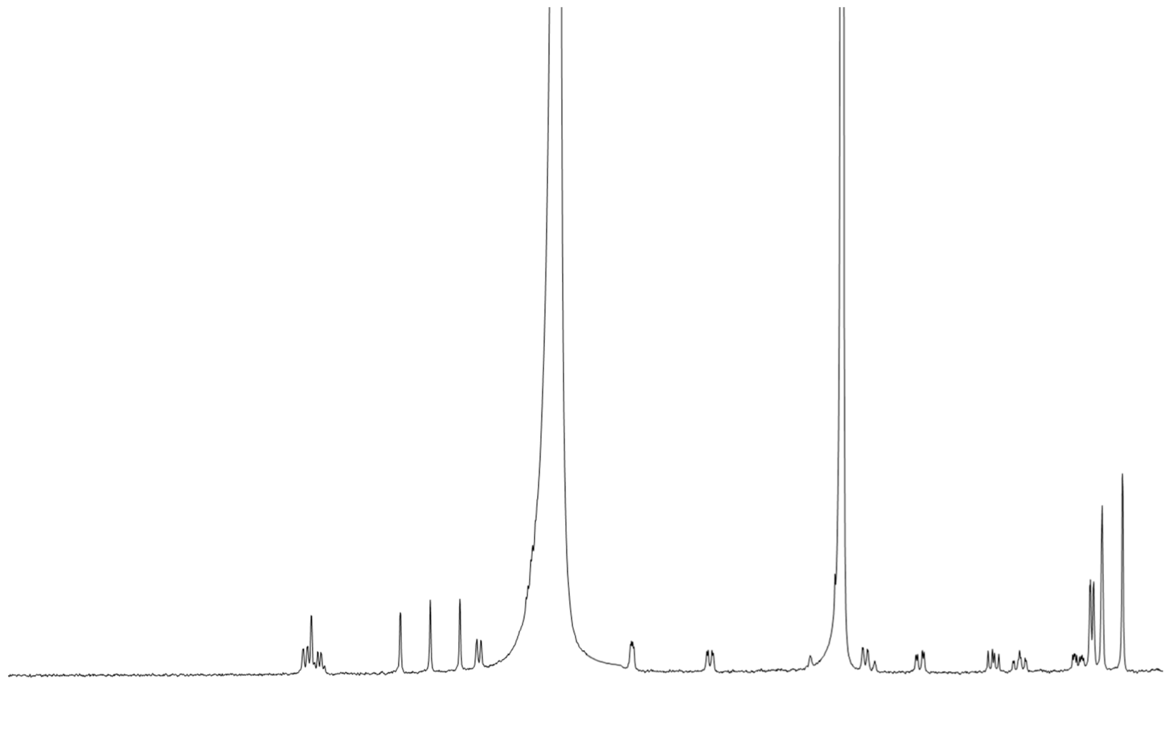


**Figure S8.** $^1$H NMR spectrum (600 MHz, $CD_3OD$) of compound **8**

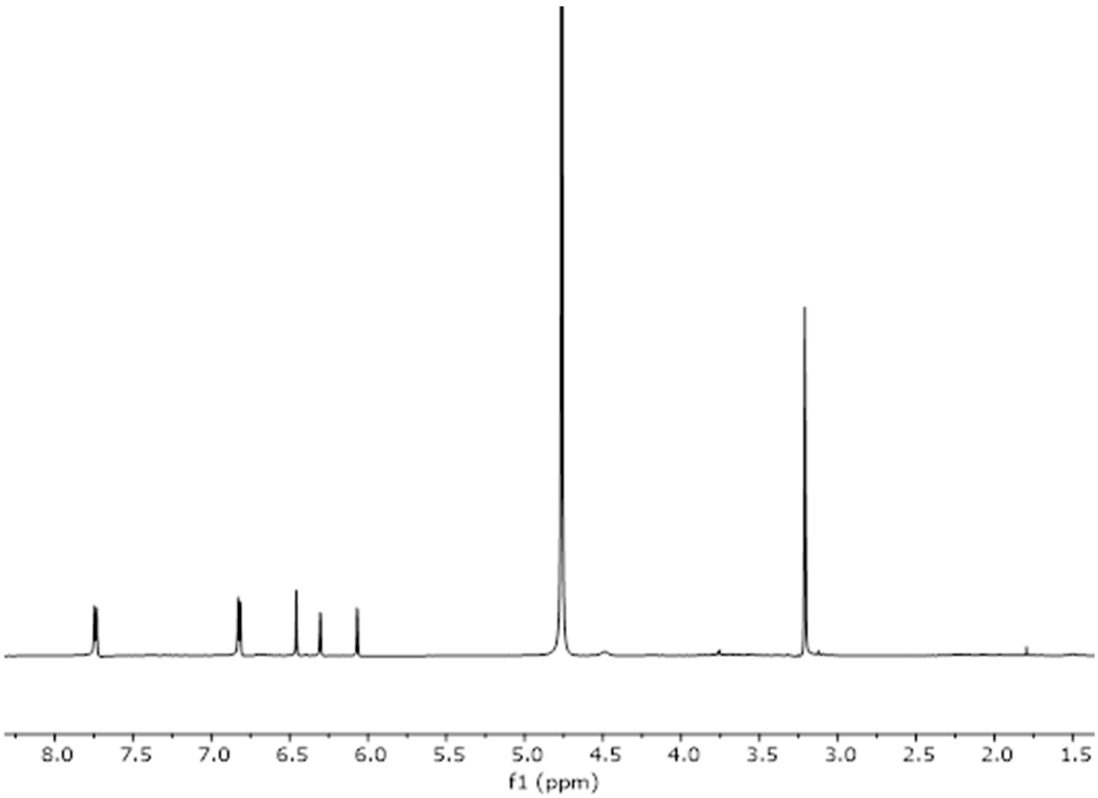


**Figure S9.** $^{1}$H NMR spectrum (600 MHz, $CD_3OD$) of compound **9**

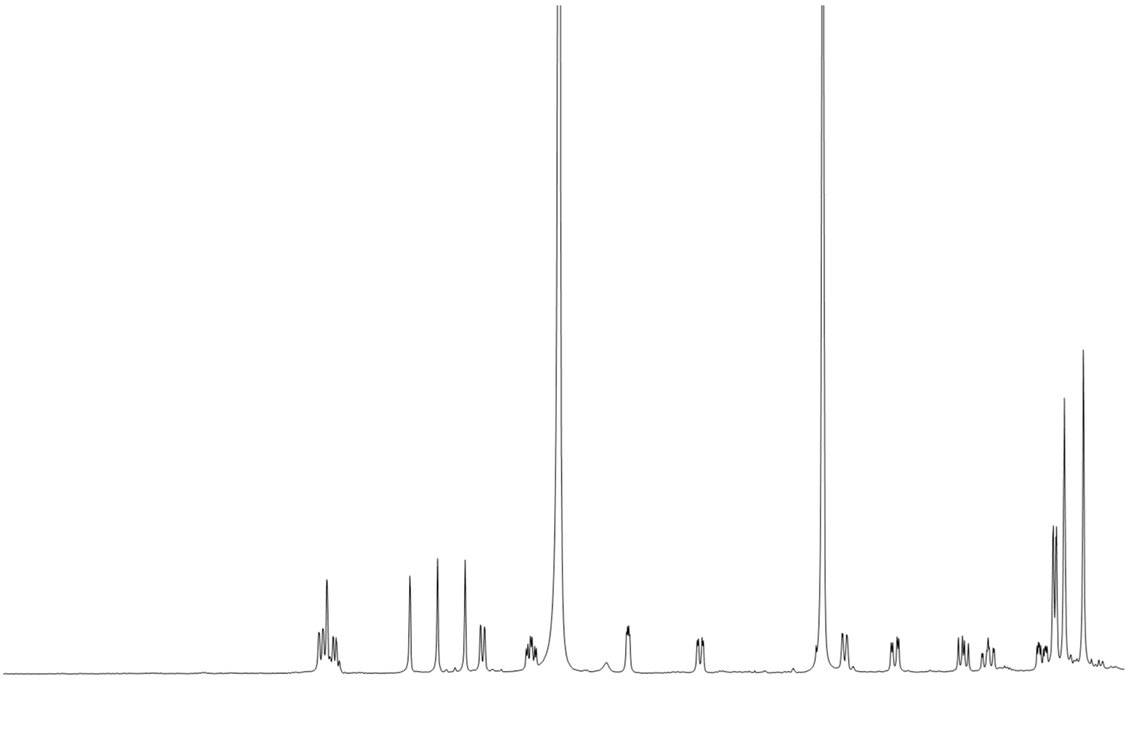


**Figure S10.** $^{1}$H NMR spectrum (600 MHz, $CD_3OD$) of compound **10**

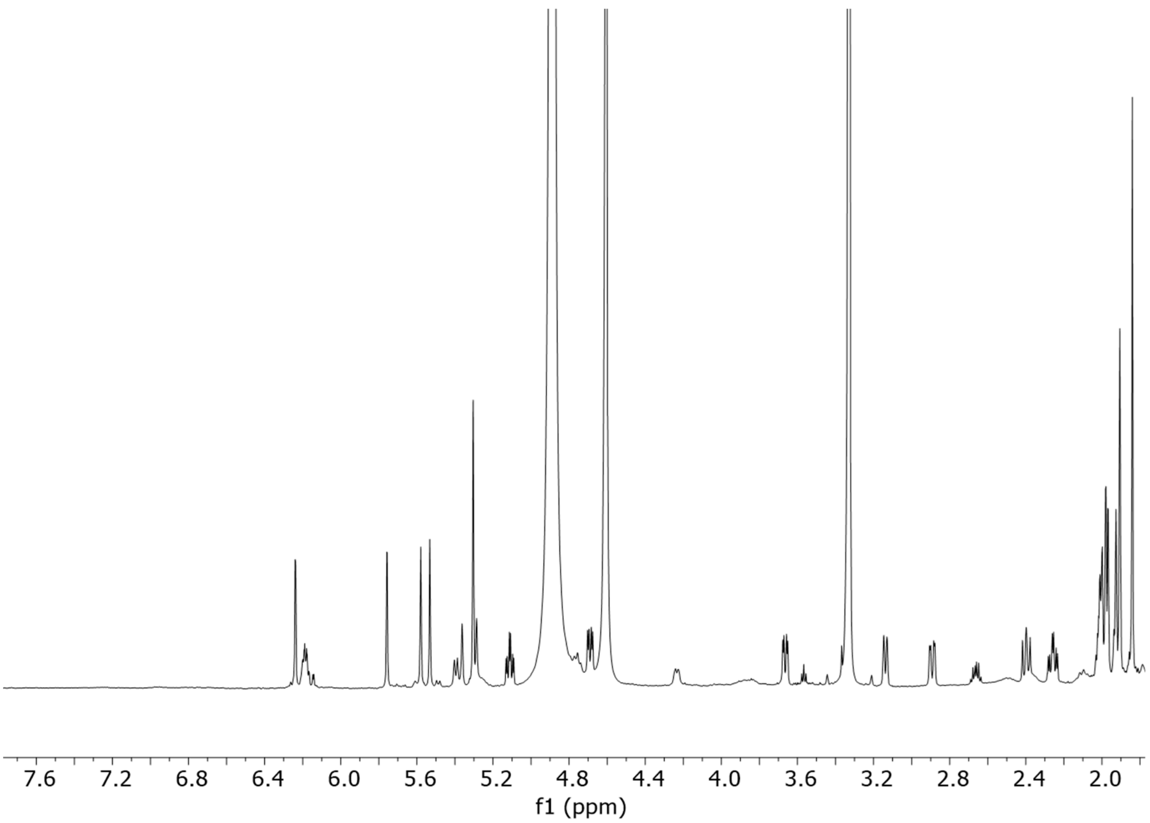


**Figure S11.** $^{1}$H NMR spectrum (600 MHz, $CD_3OD$) of compound **11**

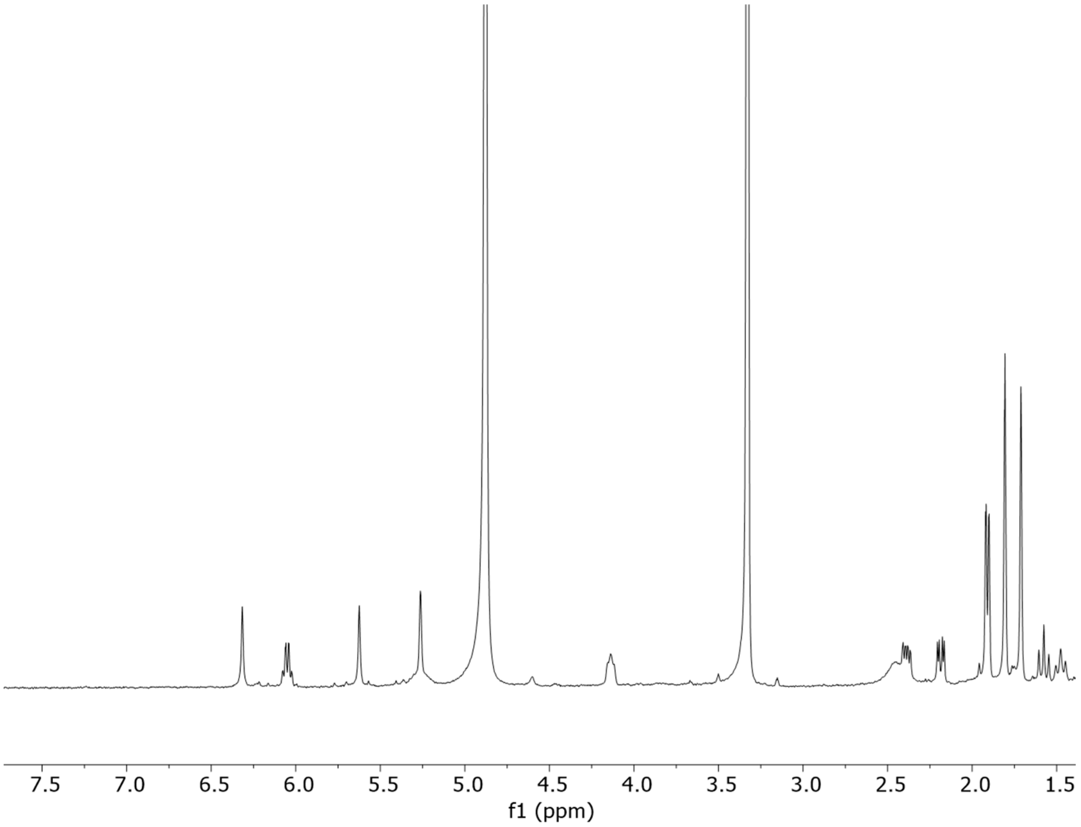


**Figure S12.** $^{1}$H NMR spectrum (600 MHz, $CD_3OD$) of compound **12**

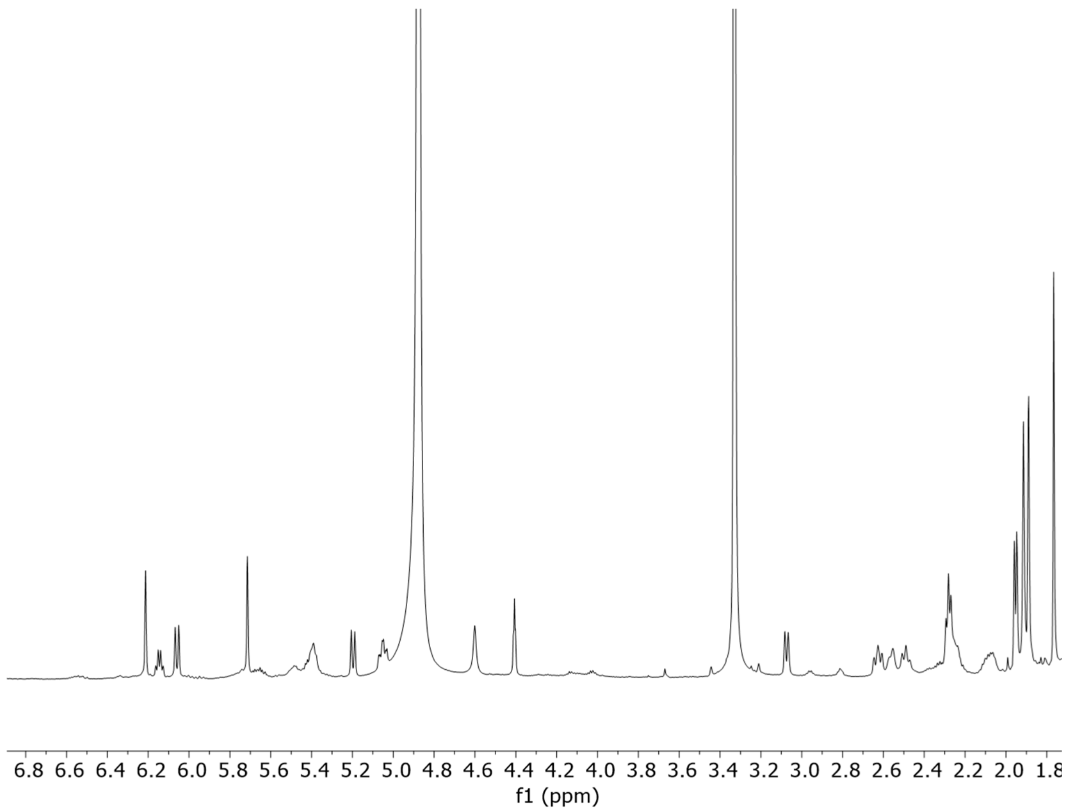


**Figure S13.** $^{1}H$ NMR spectrum (600 MHz, $CD_3OD$) of compound **14**

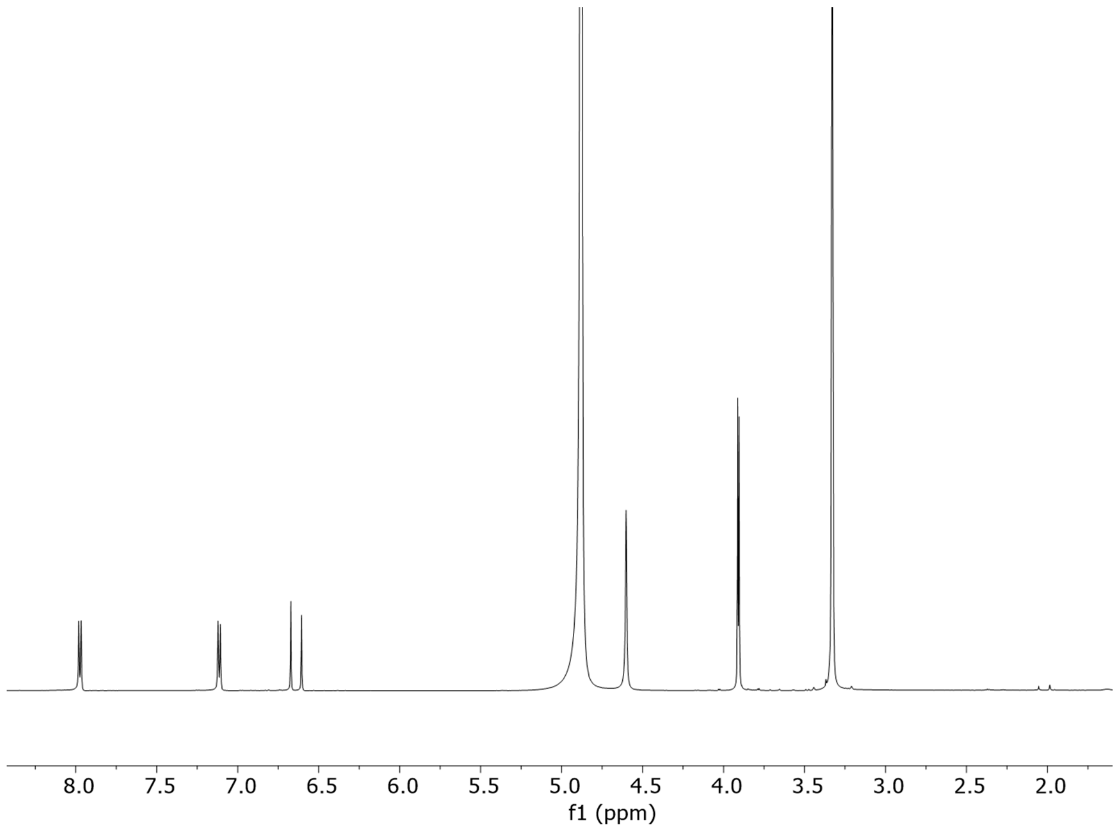


**Figure S14.** $^{1}H$ NMR spectrum (600 MHz, $CD_3OD$) of compound **15**

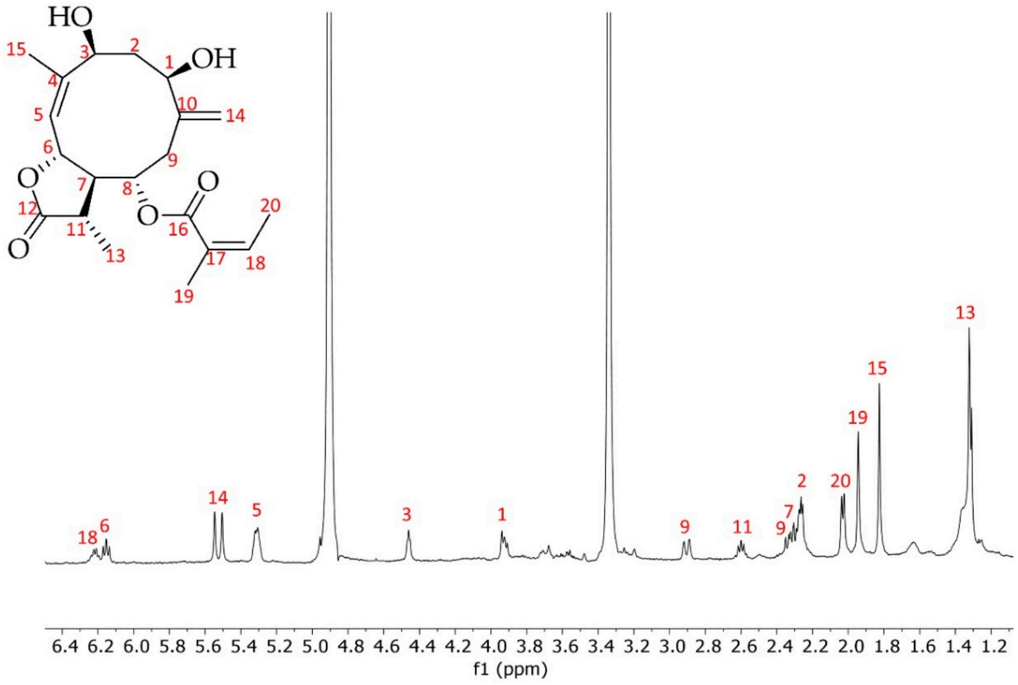


**Figure S15.** $^{1}$H NMR spectrum of compound **13**

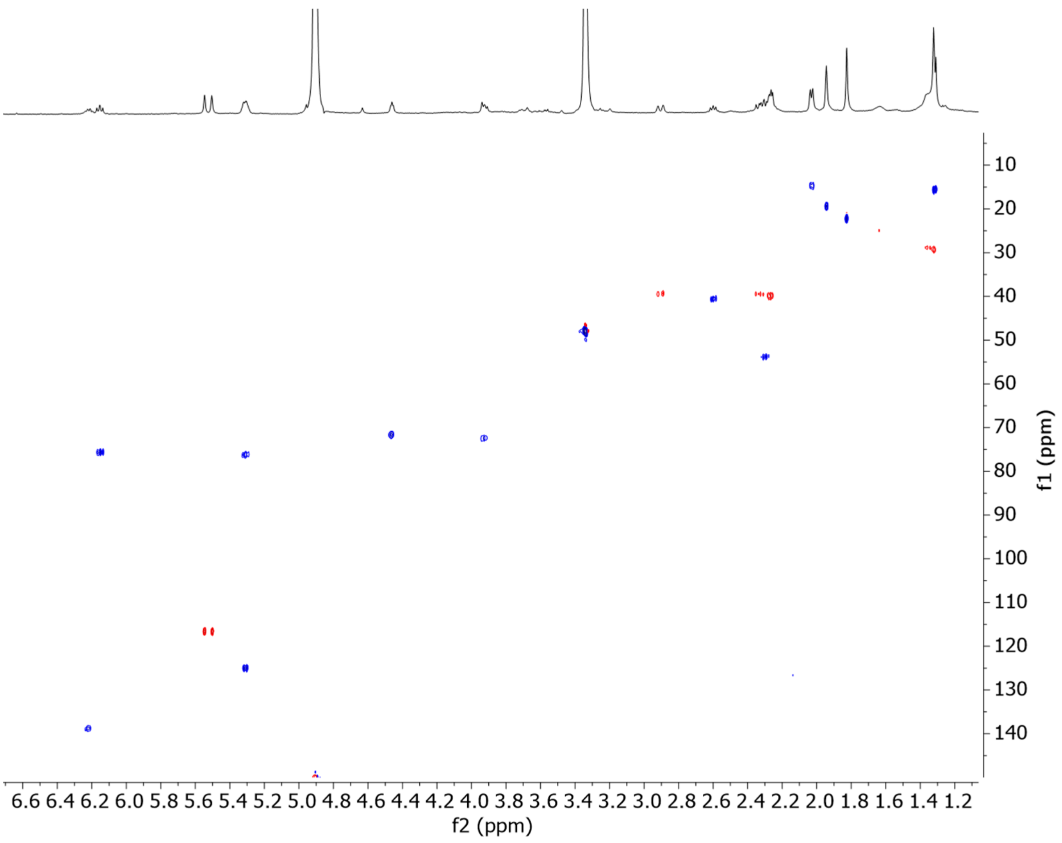


**Figure S16.** HSQC spectrum (600 MHz, $CD_3OD$) of compound **13**

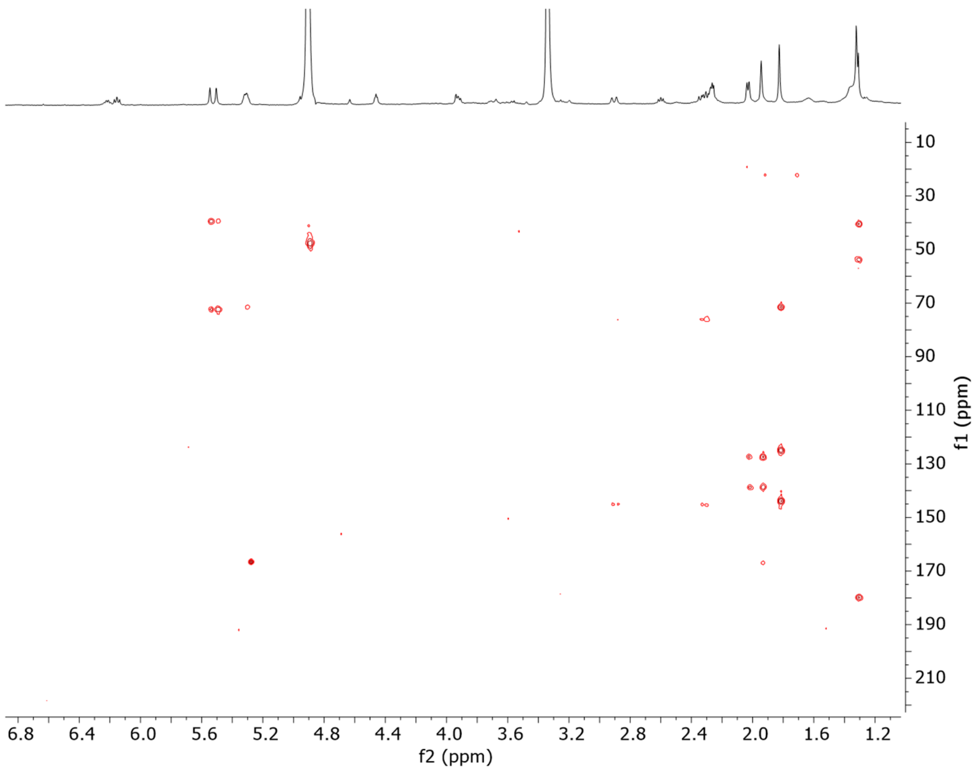


**Figure S17.** HMBC spectrum (600 MHz, $CD_3OD$) of compound **13**

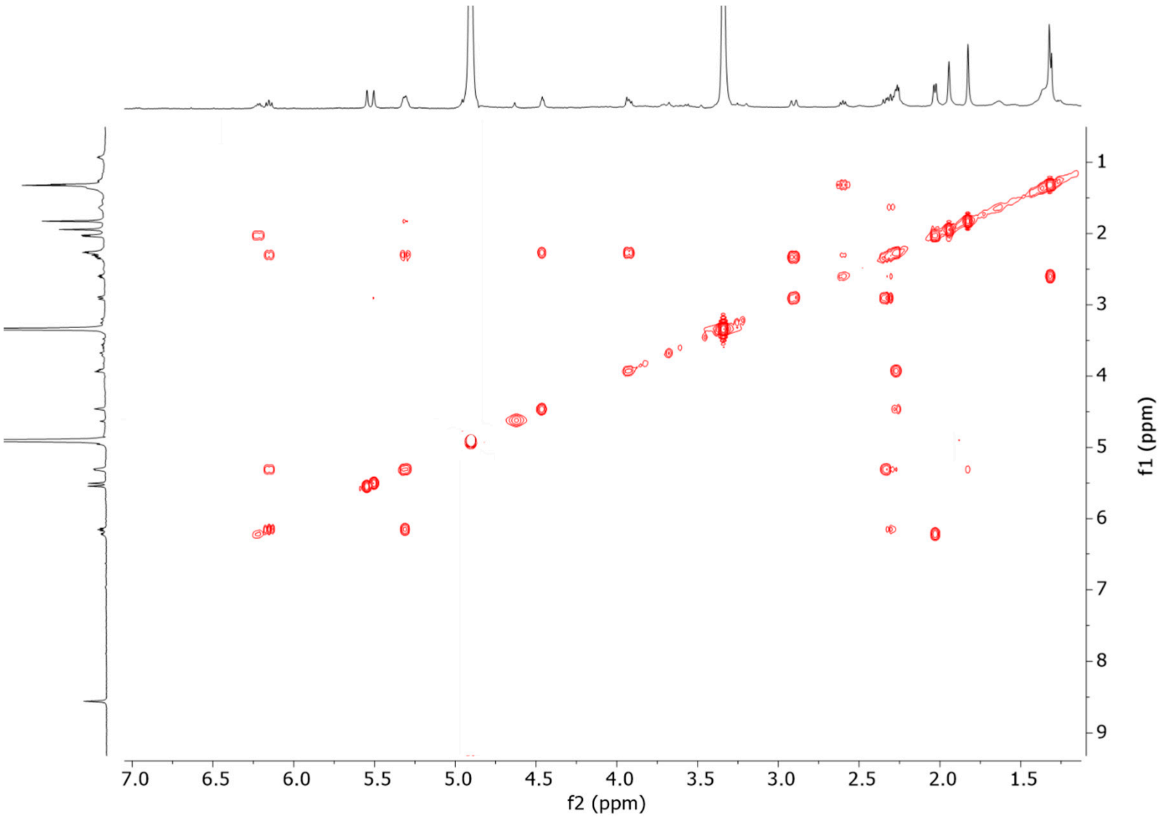


**Figure S18.** COSY spectrum (600 MHz, $CD_3OD$) of compound **13**

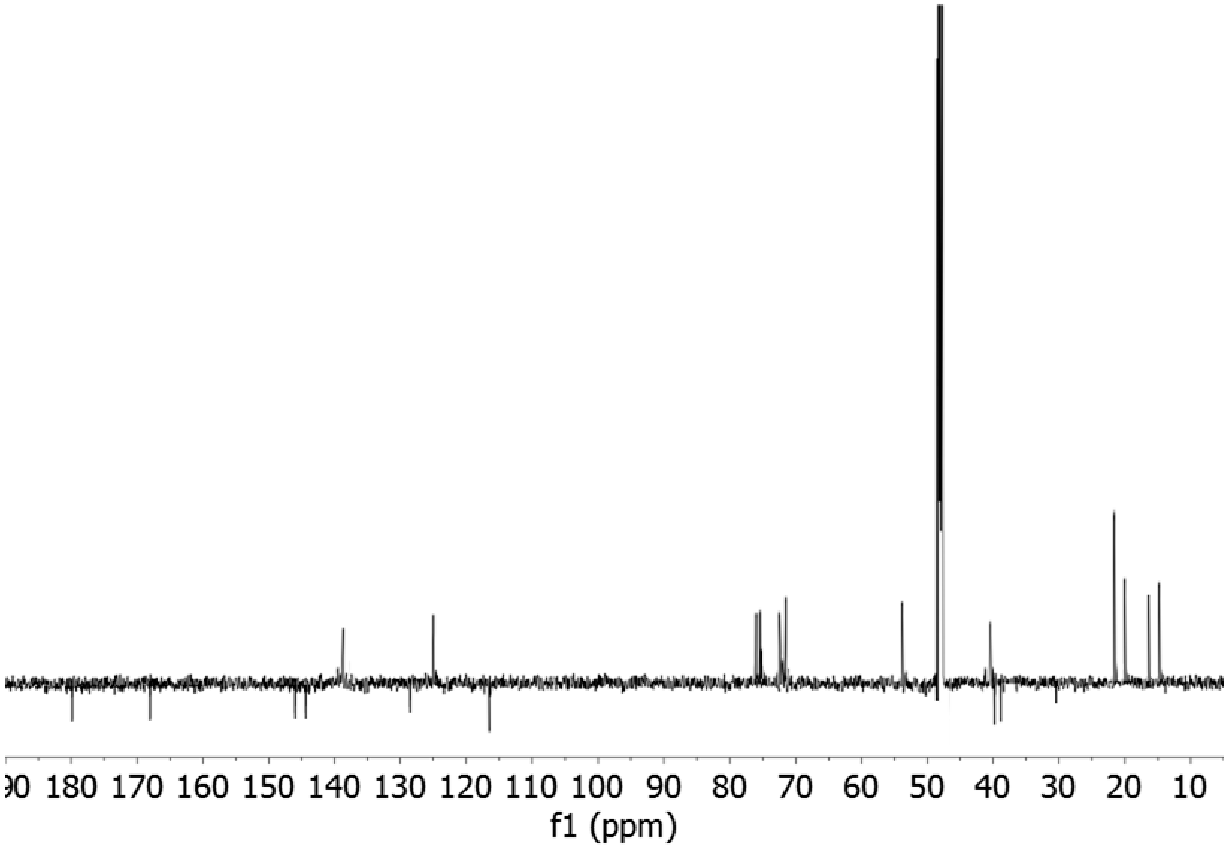

**Figure S19.** $^{13}C$ NMR spectrum (150 MHz, $CD_3OD$) of compound **13**

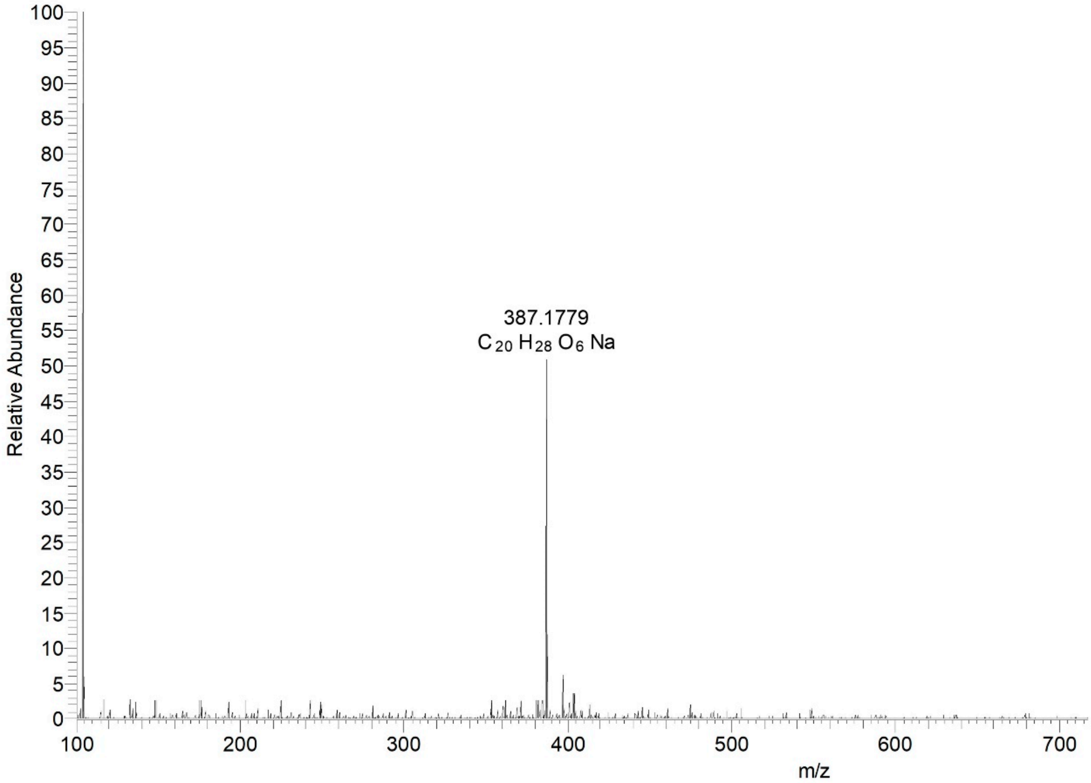


**Figure S20.** ESI-MS of compound **13**

**Table S1.** Tyrosinase inhibititory activity of specialized metabolites isolated from *C. nobile*

| **Specialized metabolites** | **$IC_{50}$±SD[a]** (μM) |
|---|---|
| **1** | 46.83 ±6.34* |
| **2** | 191.89 ±7.92** |
| **3** | 196.23 ±7.01** |
| **4** | 412.02 ±8.12** |
| **5** | 299.47 ±7.86** |
| **6** | 32.09 ±6.04* |
| **7** | 87.43 ±5.67* |
| **8** | 183.66 ±7.12** |
| **9** | 50.84 ±5.88* |
| **10** | 188.07 ±6.63** |
| **11** | 285.76 ±8.28** |
| **12** | 314.10 ±8.09** |
| **13** | 145.83 ±7.14* |
| **14** | 297.09 ±7.23** |
| **15** | 211.56 ±6.76** |
| Kojic acid | 65.53 ±8.81* |

[a] SD: Results are expressed as mean of three experiments; SD, standard deviation. *p< 0.05 ** p < 0.002 vs. control, one-way ANOVA followed by Dunnett's multiple comparison test.

**Table S2.** AchE inhibitory activity of specialized metabolites isolated from *C. nobile*

| **Specialized metabolites** | **$IC_{50}$±SD[a]** (μM) |
|---|---|
| **1** | 231.25 ±8.24** |
| **2** | 343.19 ±10.01* |
| **3** | 280.98 ±10.13* |
| **4** | 351.77 ±9.03** |
| **5** | 387.99 ±11.05** |
| **6** | 181.58 ±10.61* |
| **7** | 232.41 ±9.98* |
| **8** | 224.78 ±9.75* |
| **9** | 362.10 ±10.06** |
| **10** | 267.87±12.13* |
| **11** | 263.39 ±11.20* |
| **12** | 244.36 ±9.11* |
| **13** | 244.75 ±9.16* |
| **14** | 303.71 ±10.27** |
| **15** | 189.82 ±8.81* |
| Galantamine | 136.80 ±7.17* |

[a] SD: Results are expressed as mean of three experiments; SD, standard deviation. *p< 0.05 ** p < 0.002 vs. control, one-way ANOVA followed by Dunnett's multiple comparison test.

*UPLC-HRMSMS analysis*

LC-MS analysis was carried out on a Kinetex 2.6 μm C18 100 Å (100 × 2.1 mm) column (Phenomenex, Aschaffenburg, Germany), using a flow rate of 0.2 mL/min. A binary solvent system was used (eluent A: water with 0.1% for-mic acid (99.9:0.1, v/v), eluent B: acetonitrile with 0.1% formic acid (99.9:0.1, v/v). The HPLC gradient started at 5% B, after 30 min, % B was at 95%, this percentage was maintained for another 5 min, before returning to the starting percentage. The autosampler was set to inject 5 μL of each extract (0.5 mg/mL). ESI source parameters were the following: capillary voltage + 35 V; tube lens voltage + 50 V; ion source temperature 280 ◦C; sheath and auxiliary gas flow (N2), 12.50 and 5; sweep gas 0. The full range m/z adapted to the acquisition of MS spectra was 150–1500. For the fragmentation study, a data-dependent scan was set up through which the precursor ions corresponding to the most intensive peaks were fragmented in the MS analysis with a collision energy of 30%. Xcalibur software version 2.2 was used for instrument control, da-ta acquisition, and data analysis.

*General Experimental Procedures*

NMR spectroscopic data were acquired in MeOH-d4 (99.95%, Sigma- Aldrich) on a Bruker DRX-600 spectrometer (Bruker BioSpin GmbH, Rheinstetten, Germany) equipped with a Bruker 5 mm TCI CryoProbe at 300 K. All NMR spectra were acquired in MeOH-d4 (99.95%, Sigma-Aldrich), and standard pulse sequences and phase cycling were utilized for COSY, HSQC and HMBC spectra. Data processing for NMR experiments was carried out with Topspin 3.2 software. Main fractionation was performed over Sephadex LH-20 (Pharmacia). For purification processes, semi-preparative HPLC separations were performed on an RP-HPLC-UV system (Agilent Technologies 1260 Infinity) equipped with a binary pump (G-1312A), manual injector (G1328B), an UV detector (G-1314B) by using Sinergi 10u-Hydro RP 80A column (250 × 10.00 micron). ESI/HR/MS were carried out on Q-Exactive Classic Mass Spectrometer (Thermo Fisher Scientific, San Jose, CA, USA), operating in positive ionization mode.

*Tyrosinase inhibition assay*

30 microliters of the sample (final concentrations of 50, 100, 200 μM) and 50 μL of 100 U/mL mushroom tyrosinase were treated in 96-well plates and incubated at 37 ◦C for 15 min. Subsequently, 50 μL of 1 mM L-tyrosine was added and then reacted at 37 ◦C for 15 min. The amount of dopachrome formed was measured at 495 nm using Thermo Scientific™ Multiskan SkyHigh Microplate Spectrophotometer. Each sample has been tested in triplicate and the tyrosinase inhibitory activity was calculated using the following equation:

Tyrosinase inhibitory activity (I%) = $[1 - (S - S_0)/ (C - C_0)] \times 100$

where S is the absorbance of the sample, tyrosinase, and L-tyrosine; $S_0$ is the absorbance of the sample and L-tyrosine; C is the absorbance of tyrosinase and L-tyrosine, and $C_0$ is the absorbance of L-tyrosine (negative control). Kojic acid, a known tyrosinase inhibitor, was used as a positive control. $IC_{50}$ values were calculated through a linear regression and expressed as means ± SD (standard deviation). They were considered statistically significant with values of $p < 0.05$.

*Acetylcholinesterase inhibition assay*

The assay mechanism forecasts that the AchE enzyme hydrolyses the substrate acetylthiocholine, resulting in the product "thiocholine" which reacts with Ellman's reagent (5,5′-dithio-bis-(2-nitrobenzoic acid), DTNB) to produce 2-nitrobenzoate-5-mercaptothiocholine and 5-thio-2-nitrobenzoate (TNB); the latter can be detected at 415 nm. 150 μL of phosphate buffer (0.1 M), 20 microliters of the sample (final concentrations of 100, 200,

300 μM) and 20 μL of 0.2 U/mL AchE (from *Electrophorus electricus*) were treated in 96-well plates and incubated at 37 ◦C for 5 min. Subsequently, 30 μL of DTNB (10 mM) and 20 μL of substrate (Acetylthiocholine, 15 mM) were added and then reacted at 37 ◦C for 20 min. The amount of TNB formed was measured at 415 nm using Thermo Scientific™ Multiskan SkyHigh Microplate Spectrophotometer. Each sample has been tested in triplicate and the AchE inhibitory activity was calculated using the following equation:

AchE inhibitory activity (I%) = (1- sample absorbance /control absorbance) x 100

Galantamine, a known acetylcholinesterase inhibitor, was used as a positive control; instead, following the same protocol, buffer phosphate (20 μL) was used as negative control. $IC_{50}$ values were calculated through linear regression and expressed as means ± SD (standard deviation). They were considered statistically significant with values of $p < 0.05$.
